# Development of a web-based machine learning model for early prediction of delayed high-dose methotrexate clearance in pediatric osteosarcoma

**DOI:** 10.3389/fped.2026.1758691

**Published:** 2026-03-30

**Authors:** Fengyi Duan, Mingqing Ji, Lichun Wu, Chang Liu

**Affiliations:** 1 Pediatric Oncology, Sichuan Clinical Research Center for Cancer, Sichuan Cancer Hospital & Institute, Sichuan Cancer Center, University of Electronic Science and Technology of China, Chengdu, China; 2 Laboratory Department of Clinical Laboratory, Sichuan Clinical Research Center for Cancer, Sichuan Cancer Hospital & Institute, Sichuan Cancer Center, University of Electronic Science and Technology of China, Chengdu, China

**Keywords:** pediatric osteosarcoma, machine learning, methotrexate, delayed metabolism, prognosis

## Abstract

**Objective:**

Pediatric osteosarcoma treatment with high-dose (HD) methotrexate (MTX) poses risks of delayed clearance due to immature organ function. Interpretable machine learning facilitates proactive prediction, enabling improved monitoring and reduced toxicity risks.

**Method:**

A retrospective study (2020–2024) of 181 pediatric patients with osteosarcoma treated with HD-MTX was conducted to identify key predictors using least absolute shrinkage and selection operator (LASSO) regression and Boruta analyses. Training and test datasets were proportionally split. Ten-fold cross-validation and hyperparameter tuning were performed, followed by the development of models (LASSO, ridge, and logistic regression), which were subsequently compared. Model performance was comprehensively evaluated using metrics such as the receiver operating characteristic (ROC) and *F*1 scores.

**Results:**

Of the 181 patients, 51 experienced delayed MTX elimination. Nine predictors (MTX3H, IBil3H, Urea3H, Cr, APTT, PT, MPV, EC, and FIB) were identified. LASSO regression outperformed the other models, achieving an AUC of 0.8466, with robust performance across multiple metrics, including the ROC, *F*1 score, and decision curve analysis.

**Conclusion:**

This interpretable machine learning model effectively predicts delayed MTX elimination in pediatric patients with osteosarcoma, enhancing patient monitoring, minimizing toxicity risks, and supporting evidence-based clinical decisions. The application is publicly accessible at: https://sclslc.shinyapps.io/shiny_cls2_1model_dalex/

## 1 Introduction

Osteosarcoma is a malignant bone tumor that primarily occurs in children and adolescents, representing a significant clinical challenge. Its treatment often requires surgical resection combined with chemotherapy or supportive therapies (1–3). Among the available options, high-dose (HD) methotrexate (MTX) is widely regarded as the cornerstone of first-line chemotherapy, achieving high drug concentrations in the bloodstream and significantly improving outcomes. Authoritative clinical guidelines, including the National Comprehensive Cancer Network “Clinical Practice Guidelines for Bone Tumors,” the European Society for Medical Oncology “Guidelines for the Diagnosis, Treatment, and Follow-up of Osteosarcoma,” and the “2018 Chinese Clinical Evidence-Based Diagnosis and Treatment Guidelines for Osteosarcoma,” recommend that HD-MTX be administered intravenously at doses exceeding 8–12 g/m^2^ (2–5). This dosage is approximately three times higher than the dose typically used for other conditions, such as malignant hematological diseases, which introduces challenges, with delayed MTX clearance being a frequent clinical issue. This can cause severe hepatotoxicity, nephrotoxicity, and myelosuppression (5–9).

To address delayed MTX elimination, current clinical practice focuses on closely monitoring drug concentrations at specific intervals—24, 48, and 72 h after administration. Interventions such as calcium leucovorin rescue therapy and urine alkalization are then applied as needed to help eliminate the drug more quickly and safely. Although these measures are standard, predictions about delayed drug clearance cannot be made based on a patient's baseline characteristics or clinical signs before treatment. This lack of predictive capability highlights the urgent need for tools that can identify patients at higher risk of complications earlier in the treatment process.

Developing an accurate prediction model for delayed MTX clearance could lead to better-targeted interventions, ultimately improving patient outcomes. By using commonly collected laboratory parameters, a predictive model could provide physicians with a practical, non-invasive method for early risk assessment. This would enable more personalized care plans for children undergoing HD-MTX therapy, minimizing the likelihood of adverse reactions while preserving the drug's therapeutic benefits. Machine learning (ML) algorithms, a branch of artificial intelligence, are powerful tools for capturing complex nonlinear relationships within high-dimensional data, particularly in medical research where they are widely applied for disease prediction. Among these, least absolute shrinkage and selection operator (LASSO) regression is particularly suited for small sample datasets with high-dimensional features, as it performs automatic feature selection by shrinking irrelevant coefficients to zero, thereby reducing model complexity and minimizing overfitting, which enhances generalization. Similarly, ridge regression addresses multicollinearity among predictors and stabilizes parameter estimates, providing robust and consistent results for small and variable datasets. Logistic regression, with its simplicity, interpretability, and computational efficiency, is useful for binary classification problems, offering reliable insights and efficient training processes even in limited sample scenarios (10). SHapley Additive exPlanations (SHAP) has emerged as a state-of-the-art method for visualizing and interpreting the decision-making process of ML models. SHAP intuitively quantifies the contribution of each feature to the model's predictions, providing a clearer and more interpretable understanding of how decisions are made and avoids the limitations of the “black box” of traditional models (11).

Previous research on delayed MTX excretion has largely concentrated on acute lymphoblastic leukemia, given its higher incidence. In contrast, osteosarcoma, a less common malignancy, has received less attention despite the distinct challenges it presents. Children and adolescents with osteosarcoma receive significantly higher doses of MTX during chemotherapy, placing them at a greater risk of delayed drug clearance and associated complications. To bridge this gap, this study sought to develop a predictive model using ML techniques, complemented by a web-based tool, to enable early identification of delayed MTX elimination in clinical settings.

## 2 Methods

### 2.1 Study design and population

This retrospective study assessed MTX dosing and associated laboratory parameters in pediatric and adolescent patients with osteosarcoma treated at Sichuan Cancer Hospital between 2020 and 2024. Inclusion criteria were (1): age ≤18 years (2), confirmed diagnosis of osteosarcoma, and (3) administration of MTX-based chemotherapy during hospitalization. Exclusion criteria were (1): insufficient clinical data (defined as ≥20% missing values) and (2) concurrent malignancies other than osteosarcoma. Delayed MTX elimination was characterized by serum MTX concentrations exceeding the following thresholds at the indicated hours post-infusion: at 24 h post-infusion (MTX24H) ≥ 10.0 µmol/L, MTX48H ≥ 1.0 µmol/L, and MTX72H ≥ 0.1 µmol/L (5, 12, 13).

### 2.2 Data collection and definition

Baseline demographic variables, including age, sex, body surface area (BSA), height, and weight, and clinical characteristics such as TNM classification and MTX dosing regimen, were systematically documented. Laboratory parameters, encompassing assessments performed both prior to MTX initiation and at 3 h post-administration, were meticulously analyzed to enable a comprehensive evaluation. A detailed summary of laboratory indices is provided in Supplementary Table S1.

For variable selection, unsupervised methods were utilized to eliminate highly collinear variables, addressing multicollinearity concerns. Supervised approaches, including LASSO regression and the Boruta algorithm, were applied to analyze and screen the remaining parameters. Variables were selected based on their statistical association and relevance to delayed MTX elimination, ensuring the inclusion of clinically and biologically meaningful predictors in the model.

### 2.3 Model building and interpretation

**Figure 1:**
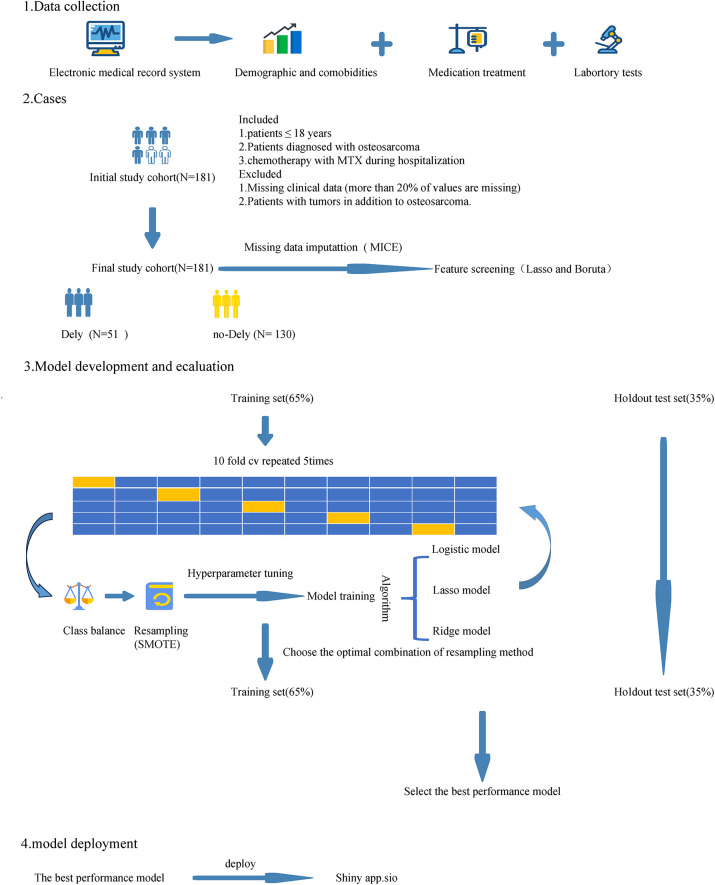
Model development process.

The model development process is illustrated in Figure 1. The dataset was randomly divided into training (65% of cases) and holdout test (35%) sets. To address missing data, we used the multivariate imputation by chained equations (MICE) method to impute missing values. Specifically, the MICE imputation model was fitted exclusively on the training set. The learned imputation models were then applied to the test set to ensure consistency across datasets and to avoid information leakage. The MICE algorithm iteratively imputes missing values for one variable at a time, using the remaining variables as predictors. This iterative process continues until the specified number of iterations is completed or convergence is achieved. For continuous variables, predictive mean matching was used to ensure that imputed values remained within the observed data range, while logistic regression was applied to impute categorical variables (14).

To address class imbalance, a hybrid resampling strategy was used, combining (Synthetic Minority Over-sampling Technique) SMOTE for minority-class oversampling and random under-sampling of the majority class. Resampling was applied only to the imputed training folds during cross-validation. The validation folds remained independent and were used solely for performance evaluation, thereby preventing information leakage and ensuring unbiased estimation of model generalization (15).

A systematic feature selection strategy was implemented to identify key predictors. First, unsupervised correlation screening was conducted to eliminate highly correlated variables, reducing redundancy and addressing multicollinearity. Next, a hybrid approach combining LASSO regression and the Boruta algorithm was employed. LASSO uses L1-norm regularization to shrink coefficients and isolate the most predictive features, while Boruta identifies important variables by comparing them against “shadow variables.” Finally, intersection analysis was performed to select features consistently identified by both methods, ensuring a cohesive and interpretable feature set while maximizing reliability and robustness.

To develop predictive models for the risk of delayed MTX excretion, we explored several algorithms, including LASSO regression, ridge regression, and logistic regression. Hyperparameter tuning for each algorithm was conducted using five rounds of 10-fold cross-validation. The final model was trained on the entire training set using the optimal combination of algorithm and resampling method, and its performance was evaluated on the holdout test set to ensure unbiased validation (16).

In clinical practice, the interpretability of ML models is crucial, as understanding the contributions of predictor variables is key to informed decision-making. To enhance interpretability, we employed SHAP, a method derived from game theory. Using the fastshap package, we calculated SHAP values to quantify the contribution of each variable to the model's predictions. These values were visualized with SHAP plots, offering clear insights into how individual predictors influenced the model's outputs. This approach helped us better understand the role of key predictors in identifying delayed MTX excretion and ensured that the model's predictions were accurate and clinically actionable (17). We developed a web-based application using the R package “shiny” to make our predictive models accessible online (18).

### 2.4 Statistical analysis

For the data characteristics, categorical variables are summarized as frequencies and percentages, while continuous variables are presented as medians with interquartile ranges. To assess differences between groups, we applied the Chi-square test for categorical variables and the Mann–Whitney *U* test for continuous variables, ensuring statistical methods were matched to the type of data being analyzed.

All data preprocessing, model training, and analyses were conducted using R software (version 4.3.1, R Foundation for Statistical Computing, Vienna, Austria). Several R packages were employed to streamline the workflow and enhance reproducibility, including DataExplorer, mice, viridis, cowplot, tidyr, tidymodels, rsample, doParallel, dplyr, recipes, skimr, ROSE, corrplot, rmda, VennDiagram, and ggplot2.

We set the significance threshold at *P* < 0.05, ensuring statistical rigor in identifying meaningful differences or patterns. This significance level was consistently applied throughout the analysis.

## 3 Results

### 3.1 Case characteristics

In our dataset (181 cases), there were 51 and 130 cases with and without metabolic delays, respectively. After proportionally dividing the dataset with a ratio of 6.5:3.5, the training set (117 cases) comprised 34 cases with metabolic delay and 83 cases without metabolic delay. In the test set (64 cases), 17 cases experienced metabolic delays and 47 cases did not.

### 3.2 Feature selection and data preprocessing

The comparison of baseline characteristics, including age, sex, height, BSA, MTX dose, and TNM classification, is summarized in Supplementary Table S2. The Mann–Whitney *U* test revealed significant differences in age, weight, height, BSA, and MTX dose. Fisher's exact test indicated significant differences in TNM classification between the two groups.

**Figure 2:**
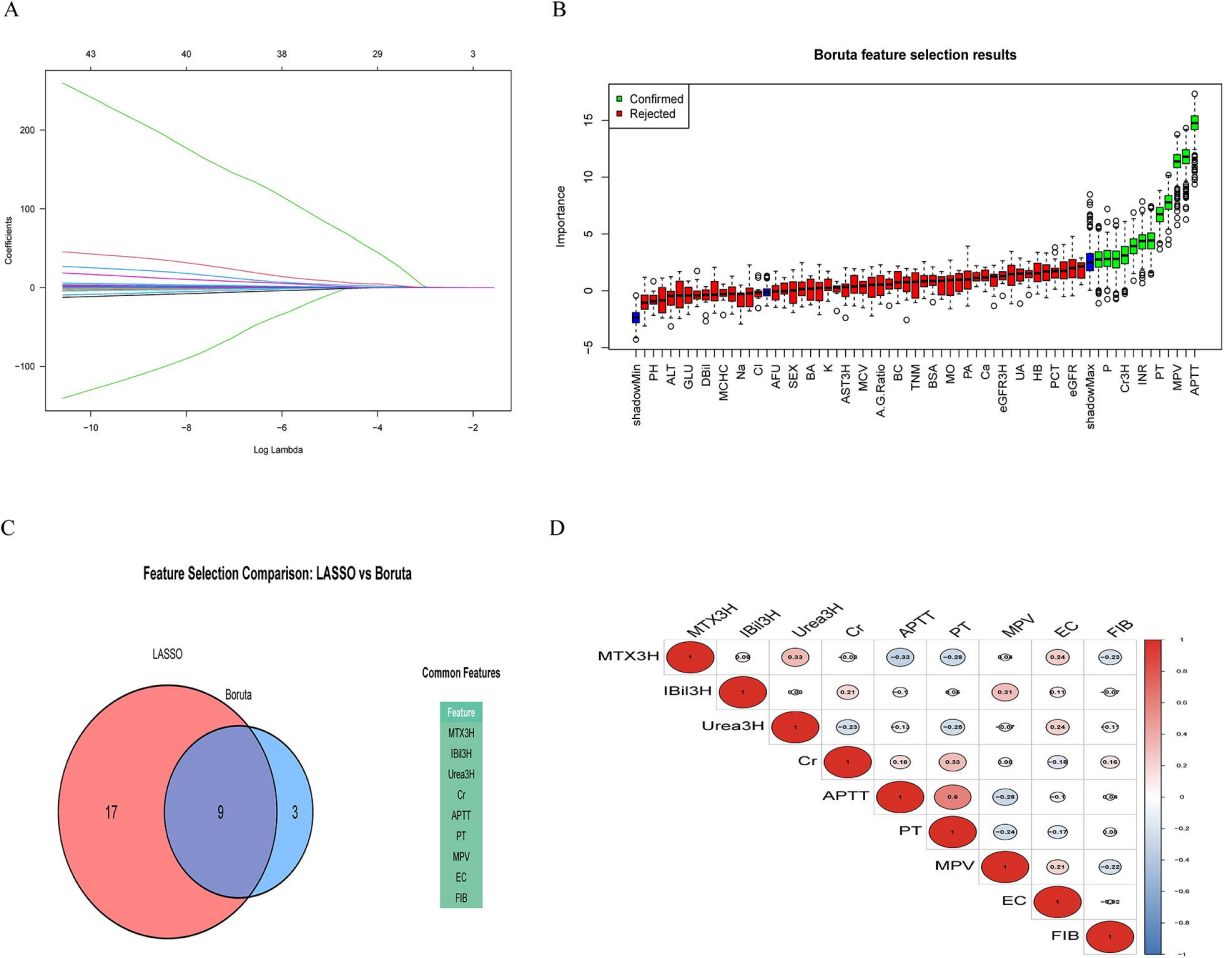
Feature selection process for identifying predictors of delayed MTX elimination. **(A)** LASSO regression analysis used to identify relevant predictors. **(B)** Boruta algorithm results showing confirmed important variables. **(C)** Intersection of variables selected by both LASSO and Boruta, yielding a final set of nine robust predictors. **(D)** Pairwise correlation analysis of the nine selected predictors, demonstrating weak inter-feature correlations and minimal multicollinearity.

To establish a reliable and interpretable predictive model, a systematic feature selection strategy integrating unsupervised and supervised methods was implemented. The process commenced with unsupervised correlation screening to eliminate highly correlated variables, thereby reducing redundancy and addressing multicollinearity at an early stage. Subsequently, a hybrid strategy combining LASSO regression and the Boruta algorithm was employed for further refinement of feature selection. To create a refined and cohesive feature set, intersection analysis was performed to identify variables consistently selected by both LASSO and Boruta (Figures 2A,B). This method ensured the final variable selection was robust and reliable. Ultimately, nine predictors strongly associated with delayed MTX elimination were identified: MTX3H, indirect bilirubin (IBil3H), Urea3H, serum creatinine (Cr), activated partial thromboplastin time (APTT), prothrombin time (PT), mean platelet volume (MPV), eosinophil count (EC), and Fibrinogen (FIB). These predictors demonstrated significant associations with the target outcome, underscoring their clinical relevance and robust predictive potential (Figure 2C). Most predictor pairs demonstrated weak correlations, highlighting the independence of these features. For example, MTX3H showed modest positive correlations with APTT. This low level of redundancy suggests that each predictor contributes unique information to the model, improving its ability to generalize.

### 3.3 Model evaluation and comparison

**Figure 3:**
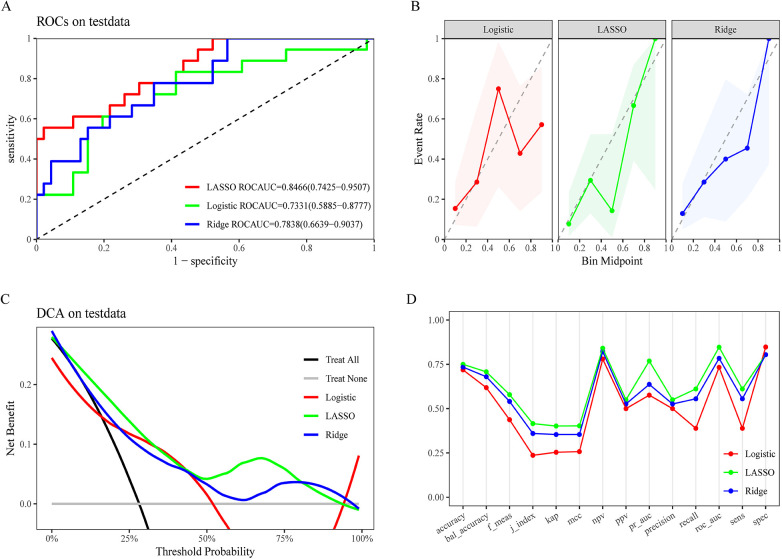
Model evaluation and comparison. **(A)** Receiver operating characteristic (ROC) curves comparing LASSO, Logistic, and Ridge models, with corresponding area under the curve (AUC) values. **(B)** Calibration plots for the three models, showing agreement between predicted and observed outcomes. **(C)** Decision curve analysis (DCA) for the test dataset, illustrating net benefit across a range of threshold probabilities. **(D)** Grouped line chart comparing multiple performance metrics among Logistic, LASSO, and Ridge models.

Delayed MTX elimination was defined as the positive class, while the absence of delayed MTX elimination was defined as the negative class. After selecting relevant variables, the dataset included serum levels of nine key indicators closely associated with the mechanism of delayed MTX elimination. Based on these features, three ML models were developed: LASSO, logistic regression, and ridge regression. The LASSO model demonstrated superior performance in predicting delayed MTX elimination. As shown in the receiver operating characteristic (ROC) curve analysis results in Figure 3A, the LASSO model achieved an AUC of 0.8466 in both the training and internal validation cohorts, outperforming the logistic regression (AUC = 0.7331) and ridge regression (AUC = 0.7838) models. Furthermore, a comprehensive comparison across various performance metrics indicated that the LASSO model consistently outperformed its counterparts in multiple indicators, such as balanced accuracy, *F*1 score, and area under the precision-recall curve (Figure 3B). The decision curve analysis further highlighted the clinical applicability of the LASSO model, which achieved the highest net benefit across a wide range of threshold probabilities compared to the logistic and ridge models (Figure 3C). The calibration analysis revealed that the LASSO model exhibited a steeper slope in medium-to-high probability ranges, indicating enhanced sensitivity in predicting delayed MTX elimination events (Figure 3D).

**Figure 4:**
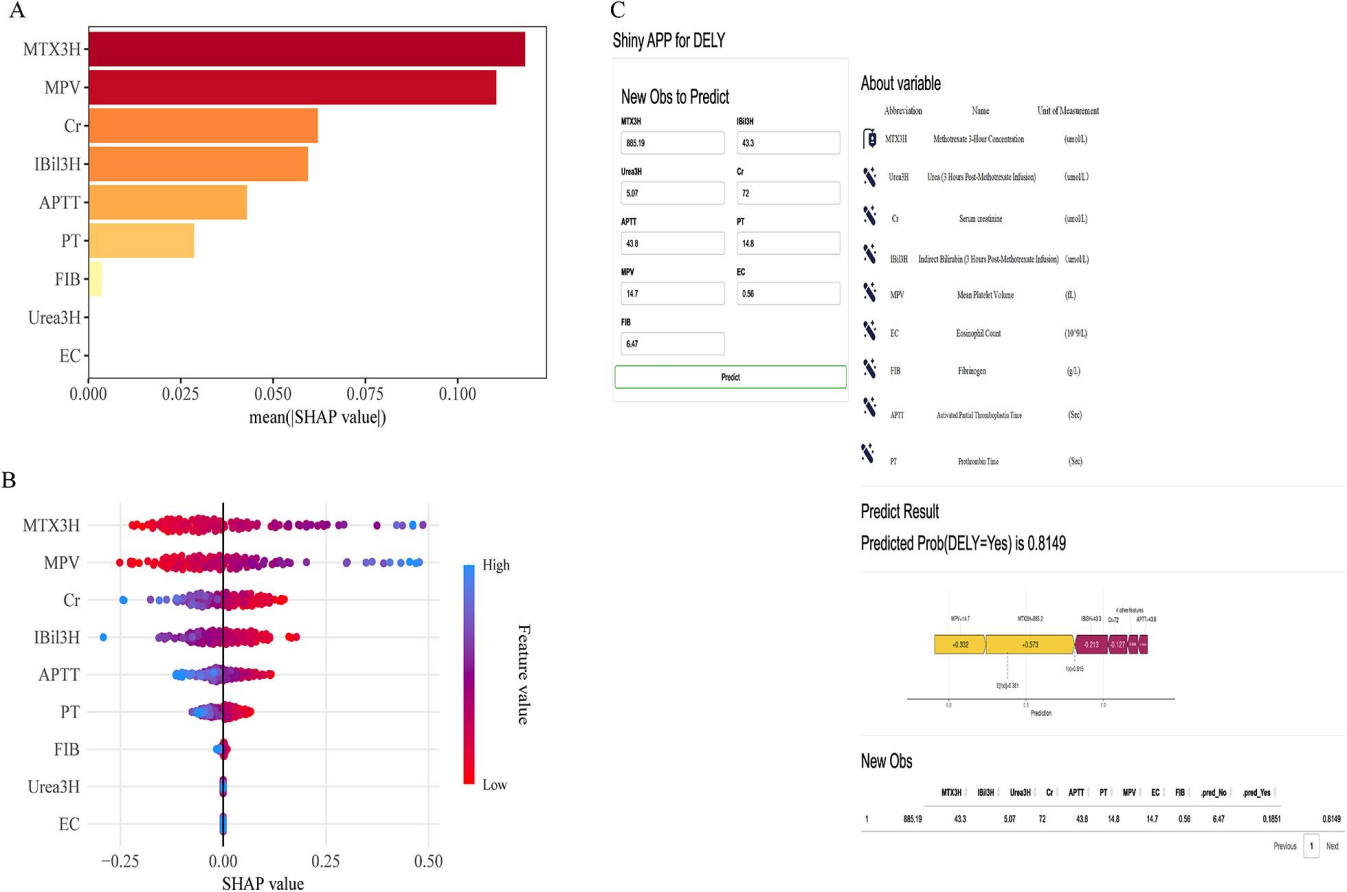
SHAP analysis and clinical prediction tool. **(A)** Bar plot of mean SHAP values for the nine selected predictors, illustrating relative feature importance. **(B)** SHAP summary plot showing the distribution of SHAP values for each variable. **(C)** Shiny application interface for DELY prediction, including input fields, variable definitions, predicted probability output, a SHAP force plot visualizing feature contributions, and a summary table of input data.

To ensure a comprehensive understanding of the selected variables, we employed the SHAP algorithm to evaluate their predictive significance in the optimal LASSO model for MTX elimination. MTX3H emerged as the most influential feature, followed by MPV, Cr, IBil3H, APTT, PT, FIB, Urea3H, and EC, reflecting their respective mean SHAP values (Figure 4A). The color coding in this figure indicates the intensity of risk, with darker red representing higher risk values and lighter shades indicating lower risks. Figure 4B further visualizes the influence of these features. This hierarchical representation underscores the critical role these nine indicators play in understanding the mechanisms of MTX elimination in osteosarcoma, thereby highlighting their potential as reliable biomarkers for clinical detection of delayed MTX elimination.

We created an interactive web-based application using Shiny to make the final prediction model accessible for individual survival forecasting and interpretation. This platform enables users to generate personalized survival predictions while offering clear insights into the factors influencing each prediction. Furthermore, it provides a global perspective of the model's overall behavior, enhancing both transparency and usability. The web application, as shown in Figure 4C, can be accessed at: https://sclslc.shinyapps.io/shiny_cls2_1model_dalex/.

## 4 Discussion

The administration of HD-MTX is associated with toxicity, and therefore, therapeutic monitoring of serum MTX concentrations is necessary during its use (19). Several adverse effects are associated with MTX toxicity levels, the most common being hepatotoxicity, neutropenia, any grade of mucositis, thrombocytopenia, and neurotoxicity. Despite adherence to the standard MTX administration protocols, most patients receiving HD-MTX experience delayed clearance, resulting in prolonged hospital stays. The traditional therapeutic drug monitoring of MTX serum concentrations and the application of suitable folinic acid rescue, coupled with proper patient hydration and urine alkalization, ensure that the occurrence of toxicity is reduced (20). However, despite MTX monitoring, rescue therapy adjustments in clinical practice rely on empirical methods. Population pharmacokinetic (popPK) and model-informed precision dosing (MIPD) approaches are grounded in mechanistic pharmacokinetic theory, and these approaches characterize drug disposition through parameterized structural models—including clearance and distribution volume—at both population and individual levels. However, their development and clinical implementation commonly depend on dense or semi-dense concentration sampling, explicit compartmental assumptions, and specialized modeling expertise and software. In real-world pediatric settings—particularly under resource constraints or when rapid decisions are required—these requirements may limit practicality. In contrast, machine learning (ML) approaches do not explicitly model underlying pharmacokinetic processes but instead learn statistical associations between routinely available clinical variables and exposure-related outcomes. This data-driven framework allows flexible representation of population heterogeneity and nonlinear relationships without prespecified (Pharmacokinetics) PK structures. Accordingly, ML-based models may facilitate the early identification of patients at risk of delayed MTX clearance with a reduced sampling burden. Importantly, such methods should be viewed as complementary screening or decision-support tools that augment, rather than replace, the established popPK and MIPD frameworks (21, 22). Currently, research on MTX-related ML models focuses on pediatric hematological malignancies. The present study developed and validated a ML-based predictive model to identify delayed MTX clearance in pediatric and adolescent patients with osteosarcoma undergoing HD-MTX chemotherapy. Among the three ML models evaluated, the LASSO regression model achieved the best performance metrics, including an AUC of 0.8446, underscoring its potential as a clinically useful tool for risk stratification. This model, which integrates nine routinely collected clinical predictors, offers an early identification method for patients at high risk of delayed MTX elimination, thereby enabling more proactive and tailored therapeutic interventions.

A key strength of our study lies in the interpretability provided by SHAP, which provided intuitive insights into the contribution of individual predictor variables for delayed MTX clearance. Our findings demonstrate that delayed MTX clearance during HD-MTX therapy is influenced by multiple factors, with SHAP analysis highlighting MTX3H (3-h MTX level) as the most impactful predictor. Interestingly, the weak correlations observed between MTX3H and MTX levels at later time points (MTX24H, MTX48H, and MTX72H) suggest that clearance dynamics are affected by additional patient-specific factors beyond early serum concentrations. These include baseline renal, hepatic, hematological, and coagulation parameters. The LASSO regression model excluding MTX3H retained a moderate discriminatory ability (AUC = 0.714), highlighting the significance of other predictors, such as MPV, Cr, IBil3H, APTT, and PT. SHAP analysis identified MPV as the second-strongest clinical correlate of HD-MTX metabolism in our pediatric osteosarcoma cohort, after MTX3H. MPV is a routinely measured index of platelet activation (23), and platelet activation has been linked to tumor progression, metastasis, and chemotherapy resistance (24–26). Although the underlying mechanisms remain speculative, activated platelets could influence MTX pharmacokinetics indirectly (for example, via the release of soluble mediators or platelet-derived microparticles) by altering the tumor microenvironment, or by modulating drug-transport pathways, any of which might affect drug distribution or clearance. SHAP showed that Cr was associated with an increased predicted risk of delayed MTX elimination, consistent with previous results (27). MTX and its metabolites are primarily eliminated via the kidneys by glomerular filtration and proximal tubular secretion, with urinary excretion as the final route (28); Cr therefore reflects renal filtration capacity, and elevated Cr is expected to reduce MTX clearance. Features related to hepatic injury and markers of hepatic synthetic function (APTT and PT) had more moderate SHAP importance, which is biologically plausible given that MTX also undergoes appreciable hepatic metabolism. Given the observational nature of our analysis, these mechanistic explanations should be viewed as hypotheses that require experimental validation. SHAP were primarily used to elucidate the relative contribution of individual variables to the model's predictions. Accordingly, the SHAP results represent statistical associations and predictive importance rather than causal relationships. Given the observational nature of our analysis, any mechanistic interpretations should be viewed as hypothesis-generating and require further experimental or prospective validation. These findings mainly serve to enhance model interpretability, while the biological relevance of the identified predictors warrants further investigation.

These findings underscore the advantage of multi-feature ML models over single-variable linear models in analyzing complex biological processes. By integrating multiple features, ML models can better capture the intricate physiological interactions that influence MTX pharmacokinetics, providing valuable insights to aid personalized treatment strategies and improve predictive accuracy in clinical settings.

Based on our findings, two key areas warrant further exploration: (1) Expanding the clinical validation phase is critical to mitigate the potential risk of overfitting due to the limited sample size and to ensure the real-world usability of the platform. Future work should involve multicenter collaboration and external validation to improve model robustness and generalizability, as well as real-world deployment enabling iterative refinement through clinician feedback and broader clinical adoption. (2) The utilization of liquid chromatography-mass spectrometry to monitor dynamic biomarkers in plasma, red blood cells, white blood cells, and platelets during MTX infusion should be explored. This approach would capture real-time pharmacokinetic and toxicodynamic changes, facilitating enhanced biomarker integration and model refinement to achieve optimized prediction accuracy and more robust clinical decision-making.

From a clinical application perspective, our web-based prediction tool tackles two key unmet needs. Firstly, it facilitates real-time risk assessment within the 3-h post-infusion window, unlike current protocols that require 24-, 48-, or 72-h monitoring (29). Secondly, the SHAP-driven interpretability framework addresses the “AI gap” in clinical oncology by offering intuitive visualizations of competing risk factors—a feature notably missing from previous MTX prediction models.

## Data Availability

The original contributions presented in the study are included in the article/Supplementary Material, further inquiries can be directed to the corresponding author.
