# Supplementary material for: Development of a web-based machine learning model for early prediction of delayed high-dose methotrexate clearance in pediatric osteosarcoma
Source: Front Pediatr. 2026 Mar 30;14:1758691. doi: 10.3389/fped.2026.1758691 (PMC13070930; doi:10.3389/fped.2026.1758691)
Supplement: Supplementary file 1 [file Table1.docx]

Supplementary Material

# Supplementary Tables

## Supplemental Table 1

| **Abbreviation** | **Full Term** |
| --- | --- |
| ALL | Acute lymphoblastic leukemia |
| APTT | Activated partial thromboplastin time |
| AUC | Area under the curve |
| BSA | Body surface area |
| Cr | Serum creatinine |
| EC | Eosinophil count |
| FIB | Fibrinogen |
| HD-MTX | High-dose methotrexate |
| IBil3H | Indirect bilirubin (3 hours post-methotrexate infusion) |
| LASSO | Least absolute shrinkage and selection operator |
| MICE | Multivariate imputation by chained equations |
| ML | Machine learning |
| MPV | Mean platelet volume |
| MTX | Methotrexate |
| MTX3H | Methotrexate serum concentration 3 hours post-infusion |
| MTX24H | Methotrexate serum concentration 24 hours post-infusion |
| MTX48H | Methotrexate serum concentration 48 hours post-infusion |
| MTX72H | Methotrexate serum concentration 72 hours post-infusion |
| PT | Prothrombin time |
| ROC | Receiver operating characteristic |
| SHAP | SHapley Additive exPlanations |
| SMOTE | Synthetic minority over-sampling technique |
| Urea3H | Urea (3 hours post-methotrexate infusion) |

Supplemental Table 2. Baseline clinical data

| **Characteristics** | **Total (n=181)** | | **Training set (n=117)** | **Validation set (n=64)** | ***P* value** |
| --- | --- | --- | --- | --- | --- |
| SEX (%) | | 181 | 117 | 64 |  |
| female | | 84 (46.4%) | 52 (44.4%) | 32 (50.0%) | 0.53 |
| male | | 97 (53.6%) | 65 (55.6%) | 32 (50.0%) |  |
| AGE (year)  (median [IQR]) | | 12.00 [9.00, 15.00] | 12.00 [9.00, 15.00] | 12.00 [7.75, 14.00] | 0.12 |
| HIGH (cm)  (median [IQR]) | | 156.00 [133.00, 162.00] | 156.00 [136.00, 164.00] | 150.00 [130.00, 162.00] | 0.14 |
| WEIGHT (kg)  (median [IQR]) | | 37.50 [27.00, 48.00] | 39.50 [28.00, 49.00] | 35.00 [26.75, 47.25] | 0.21 |
| BSA  (median [IQR]) | | 1.28 [1.02, 1.48] | 1.32 [1.06, 1.49] | 1.21 [1.01, 1.45] | 0.22 |
| Methotrexate (g)  (median [IQR]) | | 10.00 [8.00, 12.00] | 10.00 [8.00, 12.00] | 8.75 [8.00, 11.25] | 0.19 |
| DELY (%) | |  |  |  |  |
| No | | 130 (71.8%) | 83 (70.9%) | 47 (73.4%) | 0.86 |
| Yes | | 51 (28.2%) | 34 (29.1%) | 17 (26.6%) |  |
| TNM stage (%) | |  |  |  |  |
| 2 | | 4 (2.2%) | 3 (2.6%) | 1 (1.6%) | 0.86 |
| 3 | | 113 (62.4%) | 71 (60.7%) | 42 (65.6%) |  |
| 4 | | 64 (35.4%) | 43 (36.8%) | 21 (32.8%) |  |
| T stage (%) | |  |  |  |  |
| 1 | | 4 (2.2%) | 3 (2.6%) | 1 (1.6%) | 0.42 |
| 2 | | 75 (41.4%) | 47 (40.2%) | 28 (43.8%) |  |
| 3 | | 89 (49.2%) | 61 (52.1%) | 28 (43.8%) |  |
| 4 | | 13 (7.2%) | 6 (5.1%) | 7 (10.9%) |  |
| N stage (%) | |  |  |  |  |
| 0 | | 147 (81.2%) | 95 (81.2) | 52 (81.2) | 0.72 |
| 1 | | 32 (17.7%) | 20 (17.1) | 12 (18.8) |  |
| Not available | | 2 (1.1%) | 2 (1.7%) | 0 (0.0%) |  |
| M stage (%) | |  |  |  |  |
| 0 | | 128 (70.7%) | 79 (67.5%) | 49 (76.6%) | 0.60 |
| 1 | | 50 (27.6%) | 35 (29.9%) | 15 (23.4%) |  |
| 2 | | 1 (0.6%) | 1 (0.9%) | 0 (0.0%) |  |
| Not available | | 2 (1.1%) | 2 (1.7%) | 0 (0.0%) |  |
| MTX3H (µmol/L)  (median [IQR]) | | 266 [189.6, 348.5] | 269.07[196.2, 349.89] | 257.5 [182.515, 348.5] | 0.61 |
| WBC (10^9^/L)  (median [IQR]) | | 5.63 [4.01, 9.72] | 5.58 [3.99, 8.95] | 5.73 [4.2325, 10.925] | 0.55 |
| Ca (mmol/L)  (median [IQR]) | | 2.32 [2.24, 2.4] | 2.33 [2.24, 2.4] | 2.295 [2.2275, 2.42] | 0.49 |
| UCB (umol/L)  (median [IQR]) | | 15.8 [10, 20.6] | 15.5 [9.7, 20.5] | 17.05 [11.175, 20.725] | 0.36 |
| MPV (fL)  (median [IQR]) | | 9.2 [8.2, 10.1] | 9.3 [8.2, 10.1] | 9.15 [8.375, 10.325] | 0.86 |
| EC (10^9^/L)  (median [IQR]) | | 0.03 [0.01, 0.13] | 0.03 [0.01, 0.13] | 0.025 [0.01, 0.1425] | 0.86 |
| HB (g/L)  (median [IQR]) | | 106 [98, 120] | 106 [98, 119] | 105 [97, 120.25] | 0.83 |

Continuous variables are presented as median [IQR] and compared using Mann–Whitney U test; categorical variables are presented as n (%) and compared using Fisher's exact test.
